# Impact of Immunosuppressive Therapy, Vaccination, and Monoclonal Antibody Use With Outcomes in Liver and Kidney Transplant Recipients With COVID‐19: A Retrospective Study

**DOI:** 10.1002/jgh3.70072

**Published:** 2024-12-04

**Authors:** Ashley Francis, Ammad Javaid Chaudhary, Abdullah Sohail, Zahid I. Tarar, Ali Jaan, Joseph P. Cavataio, Sara Farooqui, Adarsh Varma, Syed‐Mohammed Jafri

**Affiliations:** ^1^ School of Medicine Wayne State University Detroit Michigan USA; ^2^ Department of Internal Medicine Henry Ford Hospital Detroit Michigan USA; ^3^ Department of Internal Medicine University of Iowa Hospitals and Clinics Iowa City Iowa USA; ^4^ Department of Gastroenterology and Hepatology University of Missouri Columbia Missouri USA; ^5^ Department of Internal Medicine Rochester General Hospital Rochester New York USA; ^6^ Department of Gastroenterology and Hepatology Henry Ford Hospital Detroit Michigan USA

**Keywords:** COVID‐19, immunosuppression, monoclonal antibodies, solid organ transplantation, vaccination

## Abstract

**Background and Aim:**

Patients who have undergone solid organ transplantation are at an elevated risk of severe coronavirus disease (COVID‐19) because of post‐transplantation immunosuppressive therapy. However, optimization of vaccination, modification of immunosuppression, and implementation of monoclonal antibody (mAb) therapy in transplant recipients with COVID‐19 is uncertain.

**Methods:**

A retrospective cross‐sectional study was conducted on patients who underwent liver or kidney transplants and were diagnosed with COVID‐19. The association of several vaccine doses, mycophenolate therapy, and mAB therapy with mortality outcomes after COVID‐19 diagnosis (3 and 6 months), hospitalization, and length of hospital stay were assessed.

**Results:**

This study included 255 patients with a median age of 59 (23–89) were included. Many COVID‐19 vaccine doses were not associated with any outcome; however, patients with a liver transplanted with mycophenolate had higher 3‐month (19% vs. 0%; *p* = 0.02) and 6‐month (21% vs. 0%; *p* = 0.01) mortality rates than those who did not. In addition, transplant recipients who received mAb therapy for COVID‐19 were less likely to be hospitalized (37% vs. 68%; *p* < 0.001).

**Conclusions:**

For organ transplant recipients with COVID‐19, vaccination alone may not be an optimal strategy for preventing serious outcomes. Rather, the types of organ transplant, immunosuppressive therapy (particularly mycophenolate), and COVID‐19 treatment strategy should be synergistically considered to promote an optimal therapeutic dynamic for a vulnerable population.

## 1 Introduction

COVID‐19 is a significant source of morbidity and mortality in patients with chronic diseases, particularly in those who have received solid organ transplants (SOT). Patients who have undergone SOT require postoperative immunosuppressive therapy that increases their susceptibility to infection [1, 2]. When a vulnerable population is exposed to a new and severely pathogenic virus, such as SARS‐CoV‐2, time is needed to determine the nuances of primary prevention, the proper routes of therapeutic management, and the general range of predicted morbidity and mortality.

Providing care to transplant recipients with COVID‐19 is complicated by considering whether stopping or continuing immunosuppressive therapy would lead to an optimal balance between a robust immune response against SARS‐CoV‐2 and appropriate transplant‐related immunosuppression. Thus, the combination of risk factors in transplant recipients contributes to the alarmingly high COVID‐19 mortality rate in this group, ranging from 27% to 37.8% [3, 4, 5]. Indeed, current research indicates that SOT recipients have a diminished immune response to the COVID‐19 vaccine compared with immunocompetent patients, and a study by Kwon et al. highlighted that this reduced immunogenicity in SOT recipients correlates with an increased risk of severe COVID‐19 [6]. However, other studies have presented contrasting evidence suggesting that higher vaccine doses may reduce both short‐term and overall mortality in these patients [7, 8]. Additionally, the use of monoclonal antibody (mAb) therapy has shown promising benefits for survival rates in SOT recipients who develop COVID‐19 [8, 9]. Moreover, the use of mycophenolate, a common immunosuppressive agent among SOT recipients, has shown an increased risk of developing adverse effects associated with COVID‐19 in certain transplant populations [10, 11].

In light of conflicting findings on how COVID‐19 affects transplant recipients, we conducted a retrospective study to explore the impact of vaccination status, immunosuppressive therapy (including mycophenolate therapy), and mAb therapy on outcomes in liver and kidney transplant recipients who contracted COVID‐19. Understanding how prevention and treatment strategies contribute to health outcomes in SOT recipients is critical for determining the best approaches for treating COVID‐19 in this patient population.

## 2 Methods

### 2.1 Study Population

We performed a retrospective cross‐sectional study of patients who had received liver and/or kidney transplants and were diagnosed with COVID‐19 between March 2020 and January 2022. We extracted data from the EPIC medical records at the Henry Ford Hospital in Detroit, Michigan. The inclusion criteria were recipients of transplanted liver and/or kidney, adults aged 18 years or older, and diagnosis of COVID‐19 by PCR at any point after transplantation within the study period. Individuals younger than 18 years of age, those who had not undergone organ transplantation, and transplant recipients who had not contracted COVID‐19 were excluded.

### 2.2 COVID‐19 Vaccination Status

The vaccination status was recorded as the number of doses that had been received at the time of COVID‐19 diagnosis. Patients who had received at least two doses of the mRNA vaccine BNT162b2 (Pfizer‐BioNTech) and/or mRNA‐1273 (Moderna) or one dose of the adenovirus‐vector COVID‐19 vaccine (Janssen COVID‐19) were considered fully vaccinated. Patients who had received no vaccine doses were considered unvaccinated, and those who had received only one dose were considered partially vaccinated. Patients who received a booster with either the mRNA vaccine or adenovirus‐vectored COVID‐19 vaccine were classified as having received three doses.

### 2.3 Outcomes

The primary outcomes were 3‐month and 6‐month mortality rates after COVID‐19 diagnosis. Secondary outcomes were hospitalization for COVID‐19 and length of hospital stay in days (LOS). We used hospitalization, admission to the intensive care unit, mechanical ventilation requirements, and length of hospital stay as crude markers to assess the severity of COVID‐19 illness.

### 2.4 Immunosuppressive and Monoclonal Antibody Therapies

We analyzed the use of immunosuppressants in all patients. Importantly, we only assessed data on immunosuppressants administered at the time of COVID‐19 diagnosis. In addition, we assessed the use of anti‐SARS‐CoV‐2 mAb therapy (bamlanivimab injection) to treat COVID‐19.

### 2.5 Statistical Analysis

Continuous variables were defined as mean ± standard deviation (SD) or median and interquartile range (IQR), as appropriate, and categorical variables were defined as counts and frequencies. Patient characteristics (age, sex, and race/ethnicity) and all outcomes were analyzed between groups as follows: number of vaccination doses at the time of COVID‐19 diagnosis (0 to 3 doses); patients who were receiving mycophenolate at the time of COVID‐19 diagnosis versus those who were not, and patients who received mAb therapy for COVID‐19 versus those who did not. The groups were compared using chi‐square tests for categorical variables and *t*‐tests for continuous variables. Normality was evaluated for all continuous variables, and the nonparametric Wilcoxon test was used when data were not normally distributed. Therefore, the median values were reported rather than the mean. Statistical significance was set at or equal to < 0.05. Logistic regression analysis was conducted to calculate odds ratios (ORs) for evaluating the effect of the number of COVID‐19 vaccine doses (any type) versus those not vaccinated (0 doses) on 3‐month and 6‐month mortality. Generalized linear models were used for continuous outcomes. All analyses were performed using SAS version 9.4.

## 3 Results

### 3.1 Baseline Characteristics

A total of 255 liver and kidney transplant recipients were included (68 liver only, 177 kidney only, 10 liver, and kidney). The median age was 59 years (range 23–89), and a little less than half (*n* = 108; 42%) of the patients were women (Table 1).

**TABLE 1:** Characteristics of organ transplant recipients stratified by number of vaccine doses received before COVID‐19.

		Patients by number of vaccine doses received before COVID‐19				
Characteristic	All patients *N* = 255	0 *n* = 161	1 *n* = 16	2 *n* = 56	3 *n* = 22	*p*
Age, years, median (range)	59 (23–89)	58 (24–89)	57 (30–73)	61.5 (23–88)	64.5 (33–79)	
Sex, *n* (%)						
Female	108 (42)	66 (41)	4 (25)	27 (48)	11 (50)	
Male	147 (58)	95 (59)	12 (75)	29 (52)	11 (50)	
Race/ethnicity, *n* (%)						
Asian	4 (2)	2 (1)	0 (0)	0 (0)	2 (9)	
Black	104 (41)	65 (40)	7 (44)	22 (39)	10 (45)	
Hispanic	6 (2)	6 (4)	0 (0)	0 (0)	0 (0)	
White	141 (55)	88 (55)	9 (56)	34 (61)	10 (45)	
Comorbidities, *n* (%)						
Chronic kidney disease						0.19
Stage 2	3 (1.2)	0 (0)	1 (6.3)	2 (3.6)	0 (0)	
Stage 3	5 (4)	1 (0.6)	2 (12.5)	0 (0)	2 (9)	
Stage 4	2 (0.8)	0 (0)	1 (6.3)	0 (0)	1 (4.5)	
Stage 5	0 (0)	0 (0)	0 (0)	0 (0)	0 (0)	
Chronic obstructive pulmonary disease	24 (9.4)	12 (7.5)	2 (12.5)	7 (12.5)	3 (13.6)	0.73
Congestive heart failure	17 (6.7%)	9 (5.6)	1 (6.3)	4 (7.1)	3 (13.6)	0.21
End‐stage renal disease	30 (12%)	19 (11.8)	0 (0)	7 (12.5)	4 (18)	0.91
Hypertension	90 (35.3%)	58 (36)	5 (31)	23 (41)	4 (18%)	0.87
Obstructive sleep apnea	14 (5.5%)	9 (3.52)	0 (0)	5 (9)	0 (0)	0.01
Type 2 diabetes	61 (24%)	28 (17.4)	5 (31.3)	19 (34)	9 (41%)	0.13

### 3.2 COVID‐19 Outcomes and Vaccination Status

Overall, 41 patients (16%) died within 3 months, and 45 patients (18%) died within 6 months of having COVID‐19. Of the 41 patients who died within 3 months, 37 (90%) died within 1 month of diagnosis (Table S2). The most common cause of death in patients was COVID‐19‐associated Respiratory failure (Table S5). In addition, 154 (60%) patients were hospitalized, with a median hospital LOS of 5 days (IQR, 3–12) (Table 2).

**TABLE 2:** Outcomes after COVID‐19 in organ transplant recipients by vaccine doses received.

	Patients by number of vaccine doses received before COVID‐19					
	All patients *N* = 255	0 *n* = 161	1 *n* = 16	2 *n* = 56	3 *n* = 22	*p*
3‐month mortality, *n* (%)^a^	41 (16)	25 (16)	2 (13)	12 (21)	2 (9)	0.549
6‐month mortality, *n* (%)^a^	45 (18)	29 (18)	2 (13)	12 (21)	2 (9)	0.595
Hospitalized, *n* (%)	154 (60)	98 (61)	10 (63)	34 (61)	12 (55)	0.948
Hospital LOS, days, median (IQR)	5 (3–12)	5 (3–12)	4.5 (3–8)	5 (3–9)	7.5 (5–14)	0.688

Abbreviations: IQR, interquartile range; LOS, length of stay; SD, standard deviation.

^a^ Mortality is death after COVID‐19.

Of the 255 SOT recipients, 161 (63%) had not been vaccinated, 16 (6%) had received one vaccine dose, 56 (22%) received two doses, and 22 (9%) received three doses at the time of COVID‐19 diagnosis. Table S1 illustrates the distribution of Pfizer‐BioNTech, Moderna, and Janssen vaccine doses, categorized as having been used for the first, second, or third dose. Notably, only two individuals received the adenovirus‐based COVID‐19 vaccine. The number of vaccine doses at the time of COVID‐19 infection was not significantly associated with any of the main outcomes (Table 2). Thus, if hospitalization and length of hospital stay were used as markers for COVID‐19 severity, there was no significant difference between the different vaccination groups. Logistic regression analysis showed no significant association between 3‐month or 6‐month mortality and having received any number of doses of COVID‐19 vaccine versus not having been vaccinated (Table 3).

**TABLE 3:** Logistic regression analysis of mortality after COVID‐19 based on vaccination dose status.

	3‐month mortality after COVID‐19		6‐month mortality after COVID‐19	
Vaccination status comparison	OR (95% CI)	*p*	OR (95% CI)	*p*
1 dose versus 0 doses	1.07 (0.37–3.09)	0.899	0.65 (0.14–3.02)	0.583
2 doses versus 0 doses	0.99 (0.53–1.85)	0.984	1.24 (0.58–2.64)	0.574
3 doses versus 0 doses	0.77 (0.32–1.89)	0.571	0.46 (0.10–2.06)	0.306

Abbreviations: CI, confidence interval; OR, odds ratio.

Further analysis of COVID‐19 outcomes was done to compare liver and renal transplant groups. No significant differences were seen between the two groups when comparing hospitalization or length of hospital stay, suggesting no difference in COVID‐19 severity. No difference was found between 3‐month and 6‐month mortality as well (Table S3). Out of the 45 deaths at 6 months, 37 (82%) were related to COVID‐19 infection, while 8 patients died from causes other than COVID‐19.

### 3.3 Post‐Transplant Duration and COVID‐19 Outcomes

Post‐transplant duration at the time of COVID‐19 is an important factor that may affect outcomes. In this study, the average post‐transplant duration is 6.98 (IQR 3.45, 13.16) years. When analyzing if there was a correlation between post‐transplant duration at time of COVID‐19 and the outcomes, no such statistically significant correlation was found after adjusting for vaccine dosage.

### 3.4 Immunosuppressive Therapy

Analysis of 199 patients (78%) who were taking mycophenolate versus 56 (22%) who were not taking mycophenolate showed that the use of mycophenolate was not associated with different outcomes (Table 4). However, of the 78 patients who had received liver transplantation, 53 were on mycophenolate, and 25 were not. Analysis of this subgroup showed that when considering only liver transplant recipients, mycophenolate use was associated with higher 3‐month (19% vs. 0%; *p* = 0.02) and 6‐month mortality (21% vs. 0%; *p* = 0.01) but not with hospitalization or hospital LOS (Table 5). In contrast, when analyzing patients with kidney transplants, mycophenolate use was not associated with a significant difference in mortality, hospitalization, or hospital LOS (Tables 5 and 6).

**TABLE 4:** Comparison of characteristics and outcomes by mycophenolate treatment for all organ transplant recipients who contracted COVID‐19.

	No mycophenolate *n* = 56	Mycophenolate *n* = 199	*p*
Age, years, mean ± SD	61.54 ± 14.22	56.45 ± 13.19	0.013
Sex, *n* (%)			0.026
Female	31 (55)	77 (39)	
Male	25 (45)	122 (61)	
Race/ethnicity, *n* (%)			0.109
Asian	1 (2)	3 (2)	
Black	30 (54)	74 (37)	
Hispanic	0 (0)	6 (3)	
White	25 (45)	116 (58)	
Outcomes			
3‐month mortality, *n* (%)^a^	5 (9)	36 (18)	0.099
6‐month mortality, *n* (%)^a^	6 (11)	39 (20)	0.123
Hospitalized, *n* (%)	31 (55)	123 (62)	0.383
Hospital LOS, days, median (IQR)	5 (3–12)	5 (3–12)	0.977

Abbreviations: IQR, interquartile range; LOS, length of stay; SD, standard deviation.

^a^ Mortality is death after COVID‐19.

**TABLE 5:** Comparison of outcomes by mycophenolate treatment for liver and kidney transplant recipients who contracted COVID‐19.

	No mycophenolate	Mycophenolate	*p*
Liver	*n* = 25	*n* = 53	
3‐month mortality, *n* (%)^a^	0 (0)	10 (19)	0.020
6‐month mortality, *n* (%)^a^	0 (0)	11 (21)	0.014
Hospitalized, *n* (%)	11 (44)	31 (58)	0.231
Hospital LOS, days, median (IQR)	6 (3–8)	5 (3–14)	0.276
Kidney	*n* = 35	*n* = 152	
3‐month mortality, *n* (%)^a^	5 (14)	26 (17)	0.686
6‐month mortality, *n* (%)^a^	6 (17)	28 (18)	0.860
Hospitalized, *n* (%)	22 (63)	95 (63)	0.969
Hospital LOS, days, median (IQR)	5 (4–16)	5 (3–11)	0.409

Abbreviations: IQR, interquartile range; LOS, length of stay; SD, standard deviation.

^a^ Mortality is death after COVID‐19.

**TABLE 6:** Comparison of outcomes after COVID‐19 in transplant recipients who did or did not receive monoclonal antibody therapy.

	No mAb therapy *n* = 190	mAb therapy *n* = 65	*p*
Age, years, mean ± SD	58.63 ± 13.42	54.46 ± 13.57	0.032
3‐month mortality, *n* (%)^a^	34 (18)	7 (11)	0.177
6‐month mortality, *n* (%)^a^	38 (20)	7 (11)	0.092
Hospitalized, *n* (%)	130 (68)	24 (37)	< 0.001
Hospital LOS, days, median (IQR)	5 (3–12)	6 (3–12)	0.820

Abbreviations: IQR, interquartile range; LOS, length of stay; SD, standard deviation.

^a^ Mortality is death after COVID‐19.

Analysis was also conducted for other immunosuppressants taken at the time of COVID‐19 diagnosis, including tacrolimus, everolimus, cyclosporine, and sirolimus. 77% of patients were on two immunosuppressants at the time of COVID‐19 diagnosis (Table S4). 208 patients were taking tacrolimus, 11 patients everolimus, 23 patients cyclosporine, and 7 patients mycophenolate (Tables 7, 8, 9, 10). No significant differences in outcomes were seen when comparing patients who were on the specific immunosuppressant and those who were not. Subgroup analysis separating liver and kidney transplant recipients was not conducted for these immunosuppressants.

**TABLE 7:** Comparison of characteristics and outcomes by tacrolimus treatment for all organ transplant recipients who contracted COVID‐19.

	No tacrolimus *n* = 47	Tacrolimus *n* = 208	*p*
Age, years, mean ± SD	61.38 ± 13.18	56.71 ± 13.52	0.032
Sex, *n* (%)			0.767
Female	19 (40)	89 (43)	
Male	28 (60)	119 (57)	
Race/ethnicity, *n* (%)			0.414
Asian	0 (0)	4 (2)	
Black	16 (34)	88 (42)	
Hispanic	2 (4)	4 (2)	
White	29 (62)	112 (54)	
Outcomes			
3‐month mortality, *n* (%)^a^	5 (11)	36 (17)	0.261
6‐month mortality, *n* (%)^a^	5 (11)	40 (19)	0.163
Hospitalized, *n* (%)	26 (55)	128 (62)	0.431
Hospital LOS, days, median (IQR)	4 (3–8)	5 (3–12)	0.248

Abbreviations: IQR, interquartile range; LOS, length of stay; SD, standard deviation.

^a^ Mortality is death after COVID‐19.

**TABLE 8:** Comparison of characteristics and outcomes by everolimus treatment for all organ transplant recipients who contracted COVID‐19.

	No everolimus *n* = 244	Everolimus *n* = 11	*p*
Age, years, mean ± SD	57.50 ± 13.64	59.18 ± 11.94	0.687
Sex, *n* (%)			0.681
Female	104 (43)	4 (36)	
Male	140 (57)	7 (64)	
Race/ethnicity, *n* (%)			0.340
Asian	4 (2)	0 (0)	
Black	102 (42)	2 (18)	
Hispanic	6 (2)	0 (0)	
White	132 (54)	9 (82)	
Outcomes			
3‐month mortality, *n* (%)^a^	40 (16)	1 (9)	0.519
6‐month mortality, *n* (%)^a^	44 (18)	1 (9)	0.447
Hospitalized, *n* (%)	148 (61)	6 (55)	0.685
Hospital LOS, days, median (IQR)	5 (3–12)	8 (3–20)	0.568

Abbreviations: IQR, interquartile range; LOS, length of stay; SD, standard deviation.

^a^ Mortality is death after COVID‐19.

**TABLE 9:** Comparison of characteristics and outcomes by Cyclosporine treatment for all organ transplant recipients who contracted COVID‐19.

	No cyclosporine *n* = 232	Cyclosporine *n* = 23	*p*
Age, years, mean ± SD	56.86 ± 13.62	64.70 ± 10.80	0.008
Sex, *n* (%)			0.441
Female	100 (43)	8 (35)	
Male	132 (57)	15 (65)	
Race/ethnicity, *n* (%)			0.130
Asian	4 (2)	0 (0)	
Black	99 (43)	5 (22)	
Hispanic	6 (3)	0 (0)	
White	123 (53)	18 (78)	
Outcomes			
3‐month mortality, *n* (%)^a^	40 (17)	1 (4)	0.108
6‐month mortality, *n* (%)^a^	44 (19)	1 (4)	0.079
Hospitalized, *n* (%)	142 (61)	12 (52)	0.398
Hospital LOS, days, median (IQR)	5 (3–12)	4 (3–8)	0.110

Abbreviations: IQR, interquartile range; LOS, length of stay; SD, standard deviation.

^a^ Mortality is death after COVID‐19.

**TABLE 10:** Comparison of characteristics and outcomes by sirolimus treatment for all organ transplant recipients who contracted COVID‐19.

	No sirolimus *n* = 248	Sirolimus *n* = 7	*p*
Age, years, mean ± SD	57.55 ± 13.56	58.29 ± 14.67	0.887
Sex, *n* (%)			0.454
Female	106 (43)	2 (29)	
Male	142 (57)	5 (71)	
Race/ethnicity, *n* (%)			< 0.001
Asian	4 (2)	0 (0)	
Black	102 (41)	2 (29)	
Hispanic	4 (2)	2 (29)	
White	138 (56)	3 (43)	
Outcomes			
3‐month mortality, *n* (%)^a^	40 (16)	1 (14)	0.896
6‐month mortality, *n* (%)^a^	44 (18)	1 (14)	0.813
Hospitalized, *n* (%)	151 (61)	3 (43)	0.336
Hospital LOS, days, median (IQR)	5 (3–12)	7 (3–18)	0.758

Abbreviations: IQR, interquartile range; LOS, length of stay; SD, standard deviation.

^a^ Mortality is death after COVID‐19.

### 3.5 Monoclonal Antibody Treatment

We also evaluated whether treatment with the mAb bamlanivimab was associated with outcomes after COVID‐19 in SOT recipients. Of the 255 patients, 65 (25%) received mAb therapy, and 190 (75%) did not. Among those treated with mAb, a significantly lower proportion were hospitalized (37% vs. 68%; *p* < 0.001). However, the patients who received mAb therapy did not have different mortality rates or hospital LOS (Table 6).

## 4 Discussion

In this retrospective study of transplant recipients who developed COVID‐19, we observed that while vaccination levels were not associated with differences in mortality or hospital‐associated outcomes, mycophenolate use, specifically in liver transplant patients, was associated with increased mortality, and SOT recipients who received mAb therapy were less likely to be hospitalized for COVID‐19. Our findings suggest that immunosuppression with mycophenolate may need to be adjusted in patients with COVID‐19. Additionally, mAb therapy may be particularly helpful in the generally at‐risk group of patients with SOT.

Previous studies have shown consistent trends regarding the possible benefits of a third dose of the COVID‐19 vaccine in transplant recipients. Naylor et al., in a cohort study of the effectiveness of a three‐dose vaccine regimen, found that SOT recipients had considerably lower vaccine effectiveness after two doses than the general population but that a third dose provided significantly improved effectiveness against infection and clinically important outcomes [12]. In contrast, in a case–control study examining mRNA vaccine effectiveness in immunocompromised adults hospitalized with COVID‐19, vaccine effectiveness against COVID‐19 hospitalization in SOT recipients was significantly lower than that in immunocompetent individuals [6]. While having received three doses was shown to be 48% more effective at preventing COVID‐19 hospitalization than two doses, the differences in clinical outcomes were negligible between vaccinated and unvaccinated SOT recipients already hospitalized with COVID‐19. Notably, a meta‐analysis that examined immune responses after one, two, and three vaccine doses in 11 886 transplant recipients (lung, heart, liver, and kidney) and 2125 healthy control individuals showed that transplant recipients had severely impaired immunogenic responses to COVID‐19 vaccines relative to controls, including both cellular and humoral responses [12]. However, the analysis showed that the humoral and cellular responses in transplant recipients increased by 34% and 36%, respectively, after the second dose and increased by 12% and 9%, respectively, after the third dose. Additionally, the analysis showed that immunogenicity in SOT recipients was stronger with longer time periods between transplantation and vaccination. Although the meta‐analysis did not observe any association between vaccination and clinical outcomes, the post‐transplantation time‐dependent feature of vaccine immunogenic responses is an important confounding feature that may partially explain our findings since we did not control for this time‐dependent variable. In addition, our sample included very few individuals who had received a third vaccine dose; nonetheless, trends in our study and other studies suggest that there may be at least some protective benefit of a third vaccine dose in transplant recipients, which may depend on other as‐yet‐undefined features. As fourth doses of mRNA COVID‐19 vaccines have become available and recommended for transplant recipients, further studies are needed to delineate whether boosters are helpful for patients with SOT.

Although SOT recipient immunosuppression remains a strong factor in COVID‐19‐associated morbidity, the risk of discontinuing immunosuppression during infectious illness in this population remains unclear. Among all patients, our study showed no difference in outcomes for mycophenolate, tacrolimus, everolimus, cyclosporine, and sirolimus. A meta‐analysis by Yin et al. showed similar results with tacrolimus, as its use was not associated with a higher risk of mortality or severe COVID‐19 in SOT recipients [13]. However, the results by Kolla et al. contrasted with our study. In this study, sirolimus was shown to have an increased risk of COVID‐19 hospitalization among heart transplant patients, and tacrolimus was associated with a decreased risk of hospitalization among liver transplant patients [14].

We further examined the clinical outcomes specifically associated with mycophenolate use, due to the number of studies that have evaluated its effects on COVID‐19‐related outcomes. Our study suggests that the use of this drug in patients with transplanted liver may be associated with an increased risk of death from COVID‐19, which is in contrast to certain studies that have shown improvements in outcomes with mycophenolate use [15]. In a prospective, non‐randomized, open‐label study by Sajgure et al., mycophenolate use in COVID‐19 was shown to reduce mortality and hospital duration. Within 48 h of COVID‐19 diagnosis by RT‐PCR, hospitalized patients received mycophenolate for 1 month. However, as this study did not look at transplant patients specifically, it is difficult to ascertain if these results are translatable to transplant patients hospitalized with COVID‐19 who have a greater risk of mortality compared to the general population. In contrast, studies have also linked high‐dose mycophenolate use with an impaired immune response after COVID‐19 vaccination [16, 17]. For example, in a study of patients treated at two liver transplant centers in France, Meunier et al. concluded that mycophenolate use was an independent predictor of poor antibody response after COVID‐19 vaccination [18]. However, this may not directly correlate to increased mortality because of factors such as patient‐specific comorbidities and COVID‐19 severity. Furthermore, although our study did not show similar results in kidney transplant recipients, Requião‐Moura et al. showed that mycophenolate use was associated with increased mortality from COVID‐19 in this population [10]. Similar results have also been observed in lung transplant recipients, where the administration of high‐dose mycophenolate during vaccination was independently linked to a higher incidence of breakthrough infections [11]. While it remains clear why our study showed a difference in outcomes when comparing liver and kidney transplant patients on mycophenolate, a couple of reasons can be hypothesized. First, the lack of control for comorbidities and further analysis of the cause of death (which was outside the scope of this study) remains an important reason. Without this, it remains uncertain if mycophenolate truly has a uniquely disadvantaged effect on liver transplant patients. Regardless, our observations and existing data strongly suggest that immunosuppressant regimens should be carefully selected and monitored in transplant recipients with COVID‐19 to ensure an optimal protective vaccination benefit.

Lastly, we observed that mAb therapy (bamlanivimab) was associated with a lower hospitalization rate in transplant recipients diagnosed with COVID‐19. However, other studies have shown variable results for mAb therapy in SOT recipients. For example, one study showed that in SOT recipients with mild‐to‐moderate COVID‐19, bamlanivimab monotherapy was well tolerated but not completely effective in preventing hospitalization [19]. However, in a randomized trial of patients at increased risk of severe COVID‐19, treatment with bamlanivimab and etesevimab was associated with reduced hospitalization rates and shorter LOS [20]. Owing to the rapidly rising variants of COVID‐19, the FDA revoked authorization for bamlanivimab monotherapy and authorized its combined use with etesevimab only for patients at risk of severe COVID‐19. Although the current omicron variant has stopped the authorized use of bamlanivimab/etesevimab, the use of mAb therapy in patients hospitalized with COVID‐19 should be further studied because of the potential benefits for immunocompromised groups.

Our study has several limitations. It was confined to patients treated within a single health system. Additionally, we did not adjust for variables such as the dose, type, and number of immunosuppressive therapies, the duration of immunosuppression, the difference in the severity of infection between the patients who died in 3‐month periods versus 6 months, or the presence of other comorbidities. Due to the lack of comprehensive data in the electronic medical records concerning seroconversion rates, it was not possible to ascertain the antibody response rate among our study's patient population. Moreover, there is a need for a control group comprising individuals not infected with COVID‐19 but on mycophenolate to facilitate comparative outcome analysis with those who have COVID‐19 and received mycophenolate treatment.

Our study also can be furthered to better understand COVID‐19 outcomes among liver and kidney transplant patients. In a study by Yip et al., AST/ALT elevations in patients with COVID‐19 were independently associated with increased risk of ICU admission, mechanical ventilation use, and mortality [21]. Additionally, studies have also shown that AKI might be a common consequence of COVID‐19 infection [22]. Teoh et al. discuss how AKI was associated with an increased risk of adverse events among hospitalized patients with COVID‐19. Patients with comorbidities including diabetes and abnormal liver function were also shown to be at greater risk of adverse events when found to have an AKI. These studies provide ground for furthering analysis of the outcomes collected in our study in order to expand results to include transplant patients. In addition to analyzing mortality and hospitalization data, our study can be furthered by analyzing rates of AKI and liver enzyme elevation and how they correlate to COVID‐19 outcomes in transplant patients.

## 5 Conclusion

In conclusion, although the number of vaccination doses was not associated with improved outcomes in liver and kidney transplant recipients with COVID‐19, mycophenolate use was associated with higher mortality in liver transplant recipients, suggesting that this therapy should be used with caution in the context of COVID‐19. Additionally, although mAb therapy for COVID‐19 is no longer a standard approach, we observed that SOT recipients treated with mAb were less likely to be hospitalized for COVID‐19, suggesting a potential strategy for treating COVID‐19 in certain immunocompromised groups. Overall, the poor clinical outcomes associated with COVID‐19 suggest that further therapeutic options, such as the fourth booster mRNA vaccine, are vital considerations in organ transplant recipients.

## Ethics Statement

The Ethics Committee/Institutional Review Board of Henry Ford Hospital approved the study protocol, and the study was conducted according to ethical standards.

## Consent

The authors have nothing to report.

## Conflicts of Interest

The authors declare no conflicts of interest.

## Supporting information


**Supplemental Table S1.** Vaccine types by dose order.
Supplemental Table S2. Frequency of 1 Month Mortality after COVID‐19 in Organ Transplant Recipients by Vaccine Doses Received.
Supplemental Table S3. Comparing Outcomes after COVID‐19 Between Liver and Kidney Transplant Recipients.
Supplemental Table S4. Comparing Number of Immunosuppressants* Taken at the Time of COVID‐19 Diagnosis.
Supplemental Table S5. Cause of Death (COD) Analysis.

## Data Availability

The data analyzed in this study is publicly available.
