# Supplementary material for: Impact of Immunosuppressive Therapy, Vaccination, and Monoclonal Antibody Use With Outcomes in Liver and Kidney Transplant Recipients With COVID‐19: A Retrospective Study
Source: JGH Open. 2024 Dec 4;8(12):e70072. doi: 10.1002/jgh3.70072 (PMC11617588; doi:10.1002/jgh3.70072)
Supplement: Supplementary file 1 — Supplemental Table S1. Vaccine types by dose order. Supplemental Table S2. Frequency of 1 Month Mortality after COVID‐19 in Organ Transplant Recipients by Vaccine Doses Received. Supplemental Table S3. Comparing Outcomes after COVID‐19 Between Liver and Kidney Transplant Recipients. Supplemental Table S4. Comparing Number of Immunosuppressants* Taken at the Time of COVID‐19 Diagnosis. Supplemental Table S5. Cause of Death (COD) Analysis. [file JGH3-8-e70072-s001.docx]

**Supplemental Table 1. Vaccine types by dose order**

| **Vaccine Type** | **First Doses** | **Second Doses** | **Third Doses** |
| --- | --- | --- | --- |
| Pfizer-BioNTech | 7 | 34 | 19 |
| Moderna | 7 | 22 | 3 |
| Johnson | 2 | 0 | 0 |

**Supplemental Table 2. Frequency of 1 Month Mortality after COVID-19 in Organ Transplant Recipients by Vaccine Doses Received**

|  |  | Patients by Number of Vaccine Doses Received Before COVID-19 | | | |
| --- | --- | --- | --- | --- | --- |
|  | All patients  N=255 | 0  n=161 | 1  n=16 | 2  n=56 | 3  n=22 |
| ^a^1-month mortality, n (%) | 37 (15) | 23 (14) | 1 (6) | 11 (20) | 2 (9) |

^a^Mortality is death after COVID-19

**Supplemental Table 3. Comparing Outcomes after COVID-19 Between Liver and Kidney Transplant Recipients**

|  | Kidney Transplant  n=177 | Liver Transplant  n=78 | *P* value |
| --- | --- | --- | --- |
| **Outcomes** |  |  |  |
| ^a^3-month mortality, n (%) | 31 (18) | 10 (13) | 0.347 |
| ^a^6-month mortality, n (%) | 34 (19) | 11 (14) | 0.324 |
| Hospitalized, n (%) | 112 (63) | 42 (54) | 0.156 |
| ICU admission, n (%) | 39 (22) | 10 (13) | 0.113 |
| Mechanical ventilation, n (%) | 31 (17.5) | 7 (9) | 0.141 |
| Hospital LOS, days, median (IQR) | 5 (3-12) | 5 (3-9) | 0.519 |
|  |  |  |  |

Abbreviations. IQR, interquartile range; ICU, intensive care unit; LOS, length of stay

^a^Mortality is death after COVID-19

**Supplemental Table 4. Comparing Number of Immunosuppressants* Taken at the Time of COVID-19 Diagnosis**

|  |  | Patients by Number of Immunosuppressants  (N=255) | |
| --- | --- | --- | --- |
| 0 Immunosuppressants, n (%) | 5 (2) | |  |
| 1 Immunosuppressants, n (%) | 52 (20) | |  |
| 2 Immunosuppressants, n (%) | 196 (77) | |  |
| 3 Immunosuppressants, n (%) | 2 (1) | |  |

*Immunosuppressants analyzed: tacrolimus, mycophenolate, cyclosporine, everolimus, sirolimus

**Supplemental Table 5. Cause of Death (COD) Analysis**

| **Cause of Death (COD)** | **Number of Deaths**  **(N=45)** |
| --- | --- |
| Primary COD: COVID-19 Associated Respiratory Failure | 35 |
| Other Primary COD* | 3 |
| Other COVID-19 complications without Primary Respiratory Failure** | 4 |
| Unknown | 3 |

* Hepatocellular carcinoma, Meningioma, Stage 4 lung cancer

**Multisystem organ failure, Liver failure, post-covid fibrosis and COPD
